# DABCO-Intercalated α-Zirconium Phosphate as a Latent Thermal Catalyst in the Reaction of Urethane Synthesis

**DOI:** 10.3390/molecules29235569

**Published:** 2024-11-25

**Authors:** Osamu Shimomura, Yushi Arisaka, Astrid Rahmawati, Shekh Md. Mamun Kabir, Motohiro Shizuma, Atsushi Ohtaka

**Affiliations:** 1 Department of Applied Chemistry, Osaka Institute of Technology, 5-16-1 Omiya, Ashahi-ku, Osaka 535-8585, Japanastrid.rahmawati@oit.ac.jp (A.R.); mamunkabir.butex@gmail.com (S.M.M.K.); atsushi.otaka@oit.ac.jp (A.O.); 2 Department of Wet Process Engineering, Bangladesh University of Textiles, Tejgaon, Dhaka 1208, Bangladesh; 3 Osaka Research Institute of Industrial Science and Technology, 1-6-50 Morinomiya, Joto-ku, Osaka 536-8553, Japan; shizuma@orist.jp

**Keywords:** α-zirconium phosphate, intercalation, HDI (hexamethylene diisocyanate), DABCO (1,4-diazabicyclo[2.2.2]octane), urethane, deintercalation

## Abstract

The mixture of hexamethylene diisocyanate (HDI) and butanol (BuOH) with the intercalation compound of 1,4-diazabicyclo[2.2.2]octane (DABCO) with α-zirconium phosphate (α-ZrP) has been evaluated as a latent thermal catalyst at varying temperatures. α-ZrP·DABCO did not show activity at 25 °C, but showed a high level of activity at a higher temperature of 80 °C. To clarify the reaction behavior of HDI-BuOH with α-ZrP·DABCO, a viscosity value of 1200 mPa·s·g/cm^2^ was reached at 80 °C for 30 min. To investigate the deintercalation behavior of DABCO from the α-ZrP interlayer, it was investigated in BuOH and in HDI, respectively, under heated conditions. Interestingly, XRD patterns showed a reduction in α-ZrP·DABCO for the interlayer distance due to the deintercalation of DABCO in BuOH, while no changes associated with the deintercalation of DABCO were observed in HDI. Butanol was found to be important for the deintercalation of DABCO. To examine the reactivity of bifunctional monomers, the reaction of 1,4-butanediol (1,4-BDO) and HDI with α-ZrP·DABCO were investigated to show good reactivity at 80 °C and stability at 40 °C.

## 1. Introduction

Polyurethane (PU) is the most attractive and versatile polymer, widely used in some industries such as paints, elastomers, insulators, elastic fibers, liquid coatings, furniture, and other functions [1,2,3]. Otto Bayer first discovered PU, which was obtained from aliphatic diisocyanate and glycol in 1937 at the laboratories of I. G. Farben in Leverkusen, Germany [4]. After World War II, the commercial production of PU was witnessed from toluene diisocyanate (TDI) and polyols [5]. Poly (tetramethylene ether) glycol (PTMG) was introduced by DuPont by polymerizing tetrahydrofuran based on PTMG and 4,4-diphenylmethane diisocyanate and an ethylene diamine-produced Spandex fiber called Lycra [6]. The growing interest in the simple synthesis and application protocol of PU has been increasing day by day. Various catalysts have been used to speed up the synthesis of PU. Among them tertiary amines and amidines are widely used, such as DABCO [1,4-diazabicyclo[2.2.2]octane] and DBU [1,8-diazabicyclo(5.4.0)undec-7-ene] [5,6,7]. Isocyanate compounds using PU synthesis show high reactivity with active hydrogen compounds containing catalysts. Blocked isocyanates have been used to resolve these problems [8,9,10,11]. Bantu et al. reported *N*-heterocyclic carbenes as delayed-action catalysts for PU synthesis [12,13]. Blocked isocyanates are less or not reactive with nucleophilic compounds and water, can be formed into one component with alcohols, and have a long pot life. When blocked reagents are used, the low molecular weight of deblocked compounds remains, which could affect the mechanical and thermal properties of the PU synthesis. Few researchers have explained the activation mechanism of DABCO [7].

Schwetlick et al. reported the protonation of the catalyst for the formation of urethane and the nucleophilic addition of alcohol to the isocyanate [14]. Farkas and Baker et al. reported that the complexation of isocyanate with the tertiary amines occurs first, which is followed by the nucleophilic attack of the alcohol [15,16]. Hatanaka et al. explained the basis of density functional theory (DFT) calculations on the basicity of DABCO for the dominant pathway of urethane formation [17]. Cramail and co-workers reported that for DABCO with aliphatic isocyanates such as isophorone diisocyanate, the conversion was only slightly enhanced to a non-catalyzed system. It can be explained that the catalysts that increased the basicity enhanced the catalytic activity of the isocyanate alcohol reaction [18].

An inorganic layer material α-zirconium phosphate (Zr(HPO_4_)_2_·H_2_O, α-ZrP) has been widely used for versatile and useful applications [19,20,21]. Han et al. reported that for the flame retardancy of PU, melamine cyanurate/α-ZrP (MCA@α-ZrP) was used as a hybrid flame retardant. The PU/MCA@α-ZrP nanocomposite exhibited 43.1 and 47.0% increases in the tensile strength and fracture energy, respectively [22]. Baker reported that covalently modified with epoxide to exfoliated α-ZrP was dispersed in a polymer matrix [23]. The self-assembly behavior and exfoliation of α-ZrP in PMMA matrices were achieved. In our previous study, we have reported the catalytic activity of α-ZrP·DBU in the reaction of hexamethylene diisocyanate (HDI) and phenol as a model reaction for PU synthesis [24]. In this case, the reactivity was increased by increasing the ratio of DBU/α-ZrP. Under ambient conditions, DBU is stabilized in the layers of α-ZrP, but when heated, DBU is released from the layers and shows the catalytic activity. The Lewis basicity of DABCO is lower than that of DBU, but the nucleophilicity of DABCO is higher than that of DBU [25]. DABCO as a tertiary amine catalyst is commonly used for the preparation of PU. DABCO shows high catalytic reactivity, thus it is difficult for it to reach a longer pot life. The catalytic reactivity of α-ZrP·DABCO might be interested in urethane synthesis. Furthermore, we reported that DABCO-intercalated α-zirconium phosphate (α-ZrP·DABCO) served as a latent thermal catalyst in the reaction of epoxy resins [24]. Compared with α-ZrP·DABCO and α-ZrP·DBU, α-ZrP·DABCO was stable under storage conditions at 40 °C. It is important to use stable catalysts before the heating and shows high reactivity for urethane syntheses under heating conditions. In this study, we investigated the catalytic activity of α-ZrP·DABCO in the reaction of hexamethylene diisocyanate (HDI) and butanol (BuOH) as a model reaction of PU synthesis.

## 2. Results and Discussion

**Figure 1:**
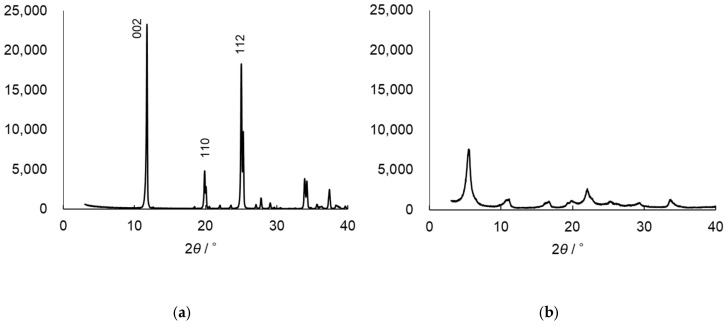
XRD patterns of (**a**) pristine α-ZrP and (**b**) α-ZrP·DABCO.

PU synthesis from diisocyanate and diol using a base catalyst is a useful quick addition reaction technique; however, maintaining the molding temperature is extremely difficult. Hence, in this study, we confirm the uniform mixing and molding temperature by using DABCO-intercalated α-zirconium phosphate as a latent thermal catalyst for PU synthesis. In our previous study, DABCO-intercalated α-ZrP (α-ZrP·DABCO) showed high stability at 40 °C and high reactivity at 140 °C in the reaction of glycidyl phenyl ether (GPE) and hexahydro-4-methylphthalic anhydride (MHHPA) [24]. The basal distance and composition was estimated from XRD and CHN elemental analysis, and the prepared intercalation compound is shown in Table 1. The composition estimated from N and H was Zr(HPO_4_)_2_·0.92DABCO·3.1H_2_O. The basal distance of the intercalation of DABCO into the interlayer of α-ZrP was 16.1 Å (2*θ* = 5.4°), expanded from that of pristine α-ZrP (7.6 Å, 2*θ* = 11.7°), as shown in Figure 1 [26]. FT-IR spectra of pristine α-ZrP and α-ZrP·DABCO are shown as Figures S1 and S2 in the Supplementary Materials.

**Figure 2:**
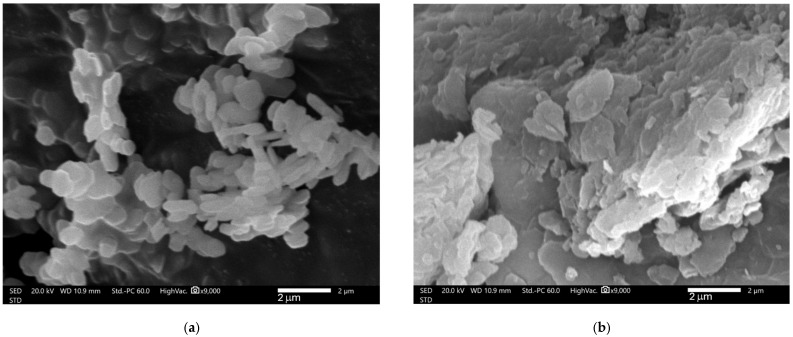
SEM images of (**a**) pristine α-ZrP and (**b**) α-ZrP·DABCO (×9000).

**Scheme 1:**
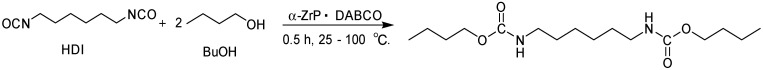
Reaction of HDI and BuOH with α-ZrP·DABCO.

**Figure 3:**
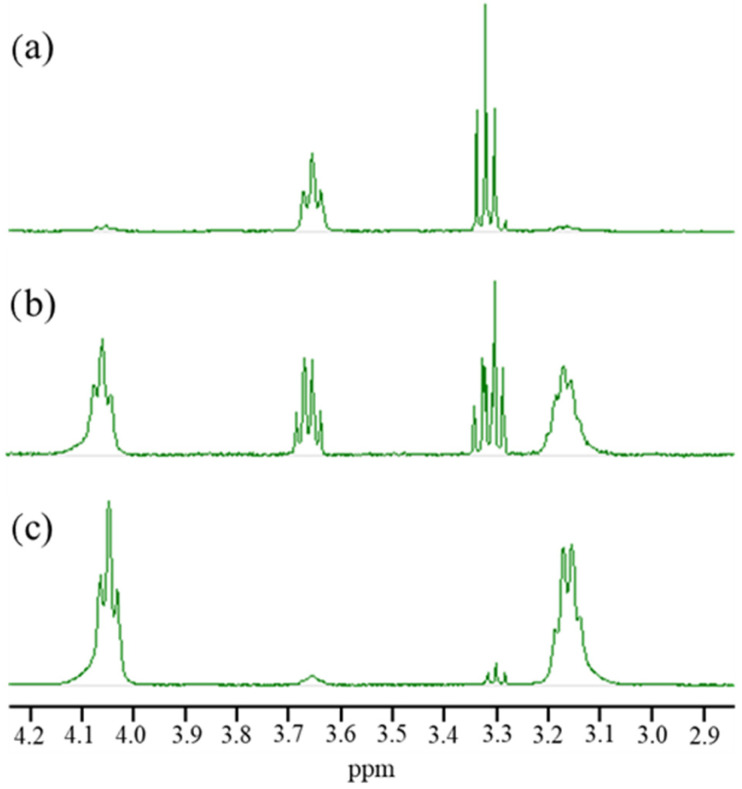
The ^1^H-NMR spectra of HDI-BuOH with α-ZrP·DABCO at (**a**) 25 °C, (**b**) 60 °C, and (**c**) 100 °C for 30 min (400 MHz, CDCl_3_ with TMS as an internal standard at 298 K).

The SEM images of (a) pristine α-ZrP and (b) α-ZrP·DABCO are shown in Figure 2. After the intercalation of DABCO, the particles were aggregated, as shown in Figure 2b. It might be due to the exfoliation of α-ZrP with the intercalation of DABCO. To investigate the catalytic activity of the α-ZrP·DABCO, the reaction of hexamethylene diisocyanate (HDI)-butanol (BuOH) with α-ZrP·DABCO was evaluated, as shown in Scheme 1. The conversion of butanol with α-ZrP·DABCO as a function of temperature was determined by ^1^H-NMR for the peak ratio of methylene protons attached to the hydroxyl group of butanol at δ3.66 and δ4.05 for the urethane product, as shown in Figure 3. The methylene protons attached to the isocyanate group of HDI at δ3.32 and δ3.16 for the urethane product (-CH_2_-NHCOO-) were observed.

**Figure 4:**
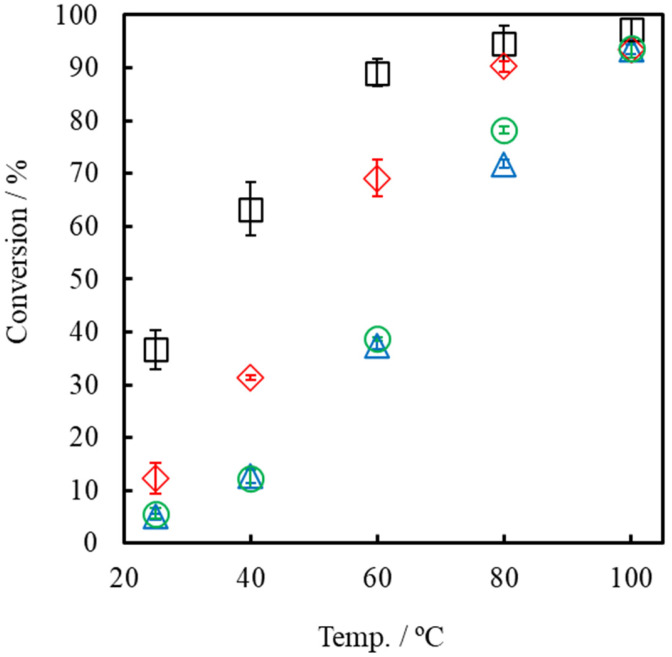
The conversion of butanol as a function of temperature during the reaction of HDI (1.0 mmol) and BuOH (2.0 mmol) with DABCO (□, 0.030 mmol), α-ZrP·DABCO (◇, 17.5 mg, DABCO content: 0.037 mmol), pristine α-ZrP (○, 19.6 mg), and no catalyst (△) for 30 min.

**Figure 5:**
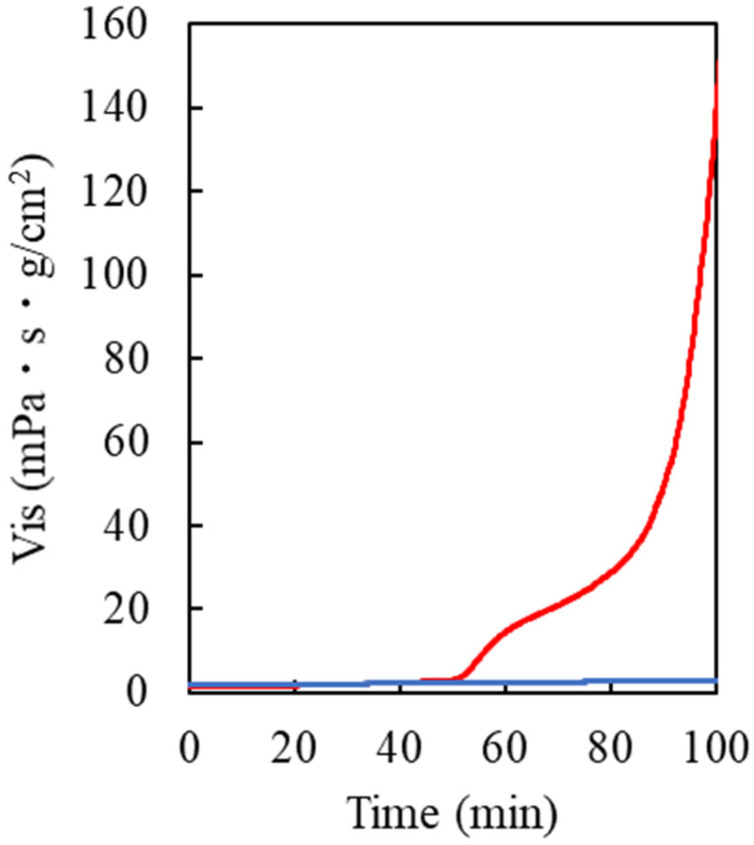
The viscosity of HDI (6.8 mmol) and BuOH (13.2 mmol) as a function of time during the reaction with DABCO (red line, 0.20 mmol), and α-ZrP·DABCO (blue line, 117 mg, DABCO content: 0.244 mmol) at 40 °C.

**Figure 6:**
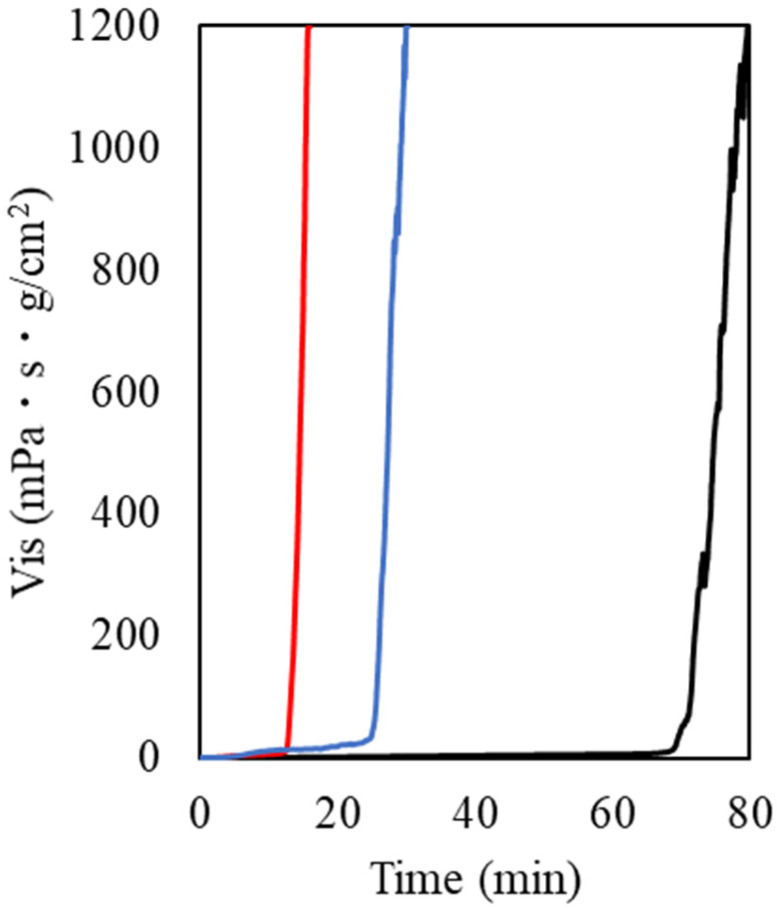
The viscosity of HDI (6.8 mmol) and BuOH (13.2 mmol) as a function of time during the reaction with DABCO (red line, 0.20 mmol), α-ZrP·DABCO (blue line, 122 mg, DABCO content: 0.254 mmol), and no catalyst (black line) at 80 °C.

Figure 4 shows the conversion of butanol (BuOH) from the reaction of HDI and BuOH after 30 min as a function of temperature during the reaction with DABCO, α-ZrP, α-ZrP·DABCO, and no catalyst. With the addition of a commercial the DABCO catalyst, the reaction proceeded even at 25 °C and the conversion was 37%. For α-ZrP·DABCO, the conversion was 12% at 25 °C; however, the conversion was 90% at 80 °C. When the temperature was raised to 100 °C, the conversion was increased to 94%. In the case of α-ZrP and no catalyst, the conversions at 80 °C were 78% and 72%, respectively. The catalyst α-ZrP·DABCO showed high reactivity under heating conditions at 80 °C and performed stabilities at 25 °C. FT-IR spectrum of the reaction product of HDI-BuOH with DABCO at 100 °C after 30 min was shown as Figure S3 in Supplementary Materials. Figure 5 shows the viscosity of HDI-BuOH as a function of time at 40 °C. With the addition of DABCO as a catalyst, the viscosity increased from 52 min; however, the addition of α-ZrP·DABCO did not increase even after 100 min. The HDI-BuOH mixture with α-ZrP·DABCO can be stored at 40 °C for at least 100 min without increasing the viscosity. Figure 6 shows the viscosity of HDI-BuOH with DABCO and α-ZrP·DABCO as a function of time at 80 °C. In the case of α-ZrP·DABCO, the viscosity increased after 25 min, whereas in the case without a catalyst, the viscosity was increased from 69 min. The initiation of the increasing viscosity was reduced by 44 min. In the case of DABCO, the viscosity was increased from 12 min which was faster than that of α-ZrP·DABCO, but the stability at 40 °C was low, as shown in Figure 5. It is important to maintain stability before heating conditions to give the time for uniform mixing and to show high reactivity under heating conditions. Alsarraf et al. reported the catalytic activity of a heterocyclic compound 7-Methyl-1,5,7-triazabicyclo[4.4.0]dec-5-ene (MTBD) that reacts with isocyanates as thermal latent catalysts in PU synthesis [27]. These catalysts show thermal latency without polymerization at 20 °C and the reaction proceeds at 60 °C for 240 min.

**Figure 7:**
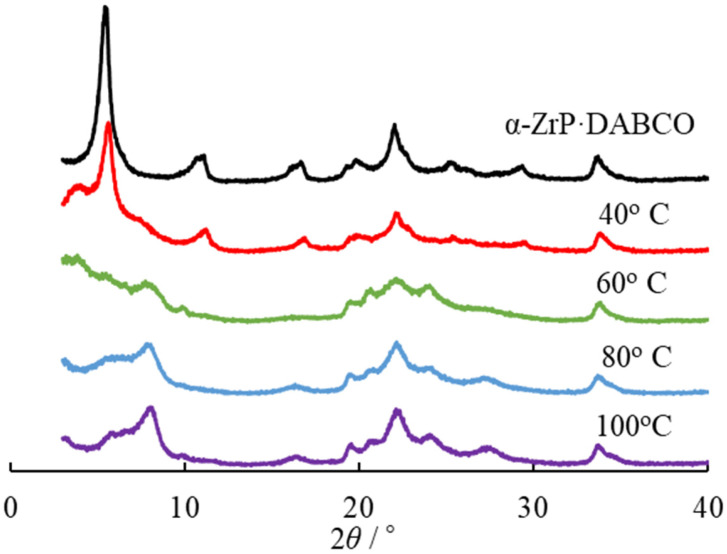
XRD patterns of α-ZrP·DABCO after the reaction of BuOH-HDI (α-ZrP·DABCO-RXN) from 40 °C to 100 °C for 30 min.

Figure 7 shows the XRD patterns of α-ZrP·DABCO after the reaction of HDI and BuOH at 40 °C to 100 °C for 30 min and washed with CHCl_3_ to remove the reacted products abbreviated as α-ZrP·DABCO-RXN. The peak was shown as the basal distance at 16.1 Å (2*θ* = 5.4°) for α-ZrP·DABCO; however, the sharp peak disappeared gradually and decreased the crystallinity, and appeared as a broad peak of 10.8 Å (2*θ* = 8.2°) for 100 °C. It can be explained that, at a higher temperature, the intercalated α-ZrP·DABCO can be deintercalated from α-ZrP layers, which enhanced the catalytic activity for the reaction of urethane synthesis.

Table 2 shows the elemental analyses of α-ZrP·DABCO after the reaction of HDI and BuOH at 40 °C to 100 °C for 30 min and washed with CHCl_3_. The increase in the C and N contents in the composition of α-ZrP·DABCO-RXN were confirmed after the reaction of HDI-BuOH.

The C and N content of α-ZrP·DABCO were 12.61 and 5.85; however, the reaction of HDI and BuOH at 40 °C shows that the C and N contents were 20.87 and 6.93. Increasing to 60 °C, the C and N contents were 33.08 and 8.59. These results show the intercalation of urethane compounds under heating conditions. If the intercalated DABCO is completely replaced by the urethane product, the N content becomes 14.6% for the C content. The N content was 27.6% for the C content for BuOH-HDI + α-ZrP·DABCO at 100 °C, as shown in Table 2, because of the remaining DABCO in α-ZrP. The thermogravimetric analysis (TGA) curve of α-ZrP·DABCO was shown as Figure S4 in the Supplementary Materials.

**Figure 8:**
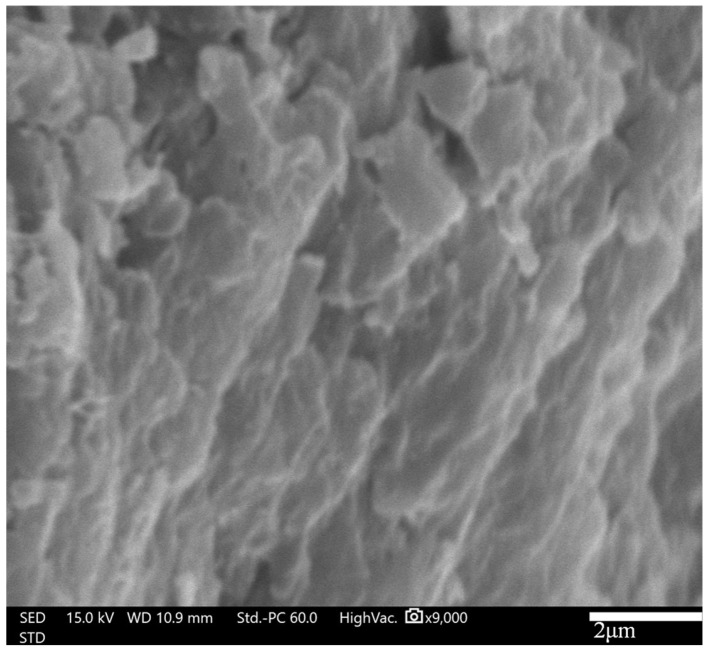
SEM image of α-ZrP·DABCO-RXN at 100 °C (×9000).

The SEM image of α-ZrP·DABCO-RXN at 100 °C is shown in Figure 8. Compared with α-ZrP·DABCO, shown in Figure 2b, the particle image was not so clear due to a reduction in the crystallinity.

**Figure 9:**
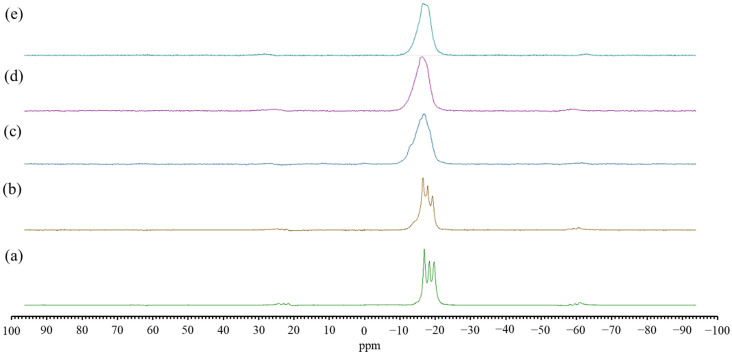
^31^P MAS NMR spectrum of (**a**) α-ZrP·DABCO and α-ZrP·DABCO-RXN at (**b**) 40 °C, (**c**) 60 °C, (**d**) 80 °C, and (**e**) 100 °C for 30 min.

The ^31^P MAS NMR spectra of the α-ZrP·DABCO reaction with HDI-BuOH at different temperatures are shown in Figure 9. In the ^31^P MAS NMR spectrum of pristine α-ZrP, the single peak was observed at δ = −20.1, as previously reported [28]. The spectra showed multiple peaks for Figure 9a α-ZrP·DABCO and Figure 9b α-ZrP·DABCO-RXN at 40 °C. The multiple peaks for the α-ZrP·DABCO compounds due to the interactions of the different positions of DABCO with the layers of α-ZrP and the interaction structures do not change at 40 °C. However, a single peak was observed for α-ZrP·DABCO-RXN at Figure 9c 60 °C, Figure 9d 80 °C, and Figure 9e 100 °C, respectively. It can be explained that at a high temperature, α-ZrP·DABCO shows the changes of the interaction of DABCO after the reaction of HDI and BuOH at 60 °C to 100 °C. We have already reported the ^31^P MAS NMR spectrum of α-ZrP·DABCO, where the single peak was observed [29]. It could affect the content of DABCO for α-ZrP.

**Figure 10:**
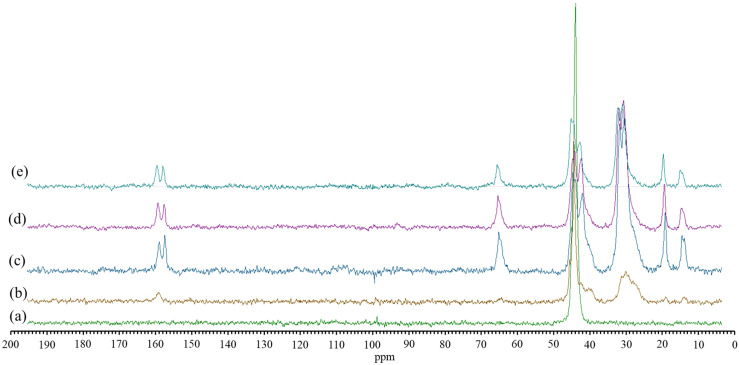
^13^C CPMAS NMR spectrum of (**a**) α-ZrP·DABCO and α-ZrP·DABCO-RXN at (**b**) 40 °C, (**c**) 60 °C, (**d**) 80 °C, and (**e**) 100 °C for 30 min.

The ^13^C CPMAS NMR spectra of the α-ZrP·DABCO reaction in HDI-BuOH at different temperatures are shown in Figure 10. The ^13^C CPMAS NMR spectrum showed α-ZrP·DABCO peaks at δ 45.1. The carbonyl carbon peaks -NH-COO- for the HDI-BuOH reaction products were at δ 159.6 and 157.9, and the methylene carbon -COO-CH_2_- peaks at δ 65.6 were clearly observed in Figure 10c–e. Therefore, in the reaction of HDI and BuOH with α-ZrP·DABCO, the urethane products were intercalated into the interlayer of α-ZrP.

**Figure 11:**
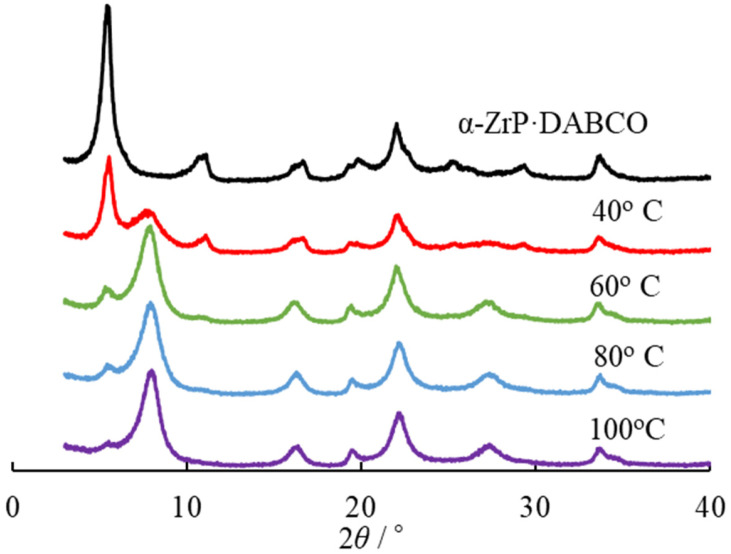
XRD patterns of α-ZrP·DABCO after the reaction of BuOH from 40 °C to 100 °C for 30 min.

Figure 11 shows the interlayer behavior of α-ZrP·DABCO treated with BuOH under heating conditions; therefore, XRD patterns of α-ZrP·DABCO were treated with BuOH at 40 °C to 100 °C for 30 min. The peak was shown as a basal distance at 16.1 Å (2*θ* = 5.4°) for α-ZrP·DABCO; however, the sharp peak gradually disappeared and a broad peak of 11.2 Å (2*θ* = 7.9°) was observed for 100 °C. It can be explained that, at a higher temperature, the intercalated α-ZrP·DABCO can be the deintercalation of DABCO from α-ZrP layers, which enhances the catalytic activity.

**Figure 12:**
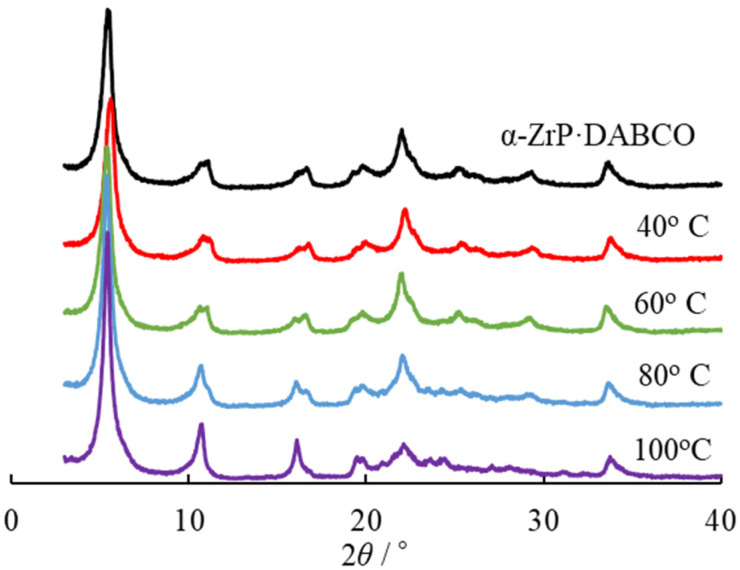
XRD patterns of α-ZrP·DABCO after the reaction of HDI from 40 °C to 100 °C for 30 min.

The XRD patterns of α-ZrP·DABCO treated with HDI at 40 °C to 100 °C for 30 min are shown in Figure 12. The peak is shown as the basal distance at 16.1 Å (2*θ* = 5.4°) for α-ZrP·DABCO. Most importantly the basal distance did not change at 40 °C: 15.7 Å (2*θ* = 5.6°), 60 °C: 16.3 Å (2*θ* = 5.4°), 80 °C: 16.4 Å (2θ = 5.4°), or 100 °C: 16.3 Å (2*θ* = 5.4°). It can be explained that at a higher temperature, the intercalated α-ZrP·DABCO cannot change with HDI, implying that there is no deintercalation effect of DABCO from α-ZrP. The deintercalation behavior might be influenced by the monomer size, functional groups, and polarity.

Table 3 shows the elemental analyses of α-ZrP·DABCO after the treatment of BuOH from 40 °C to 100 °C for 30 min. The *N*-contents were decreased from 5.85% of α-ZrP·DABCO to 4.73% after the treatment of BuOH at 100 °C because of the deintercalation of DABCO.

**Figure 13:**
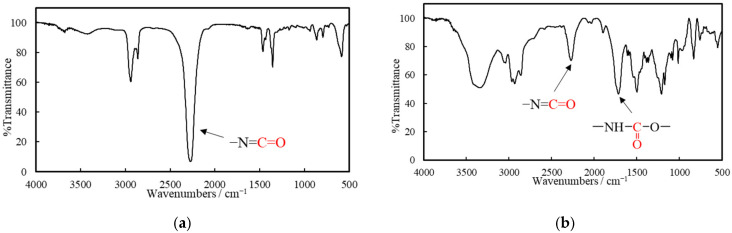
FT-IR spectra of (**a**) just-mixed 1,4-BDO and HDI and (**b**) stirred 1,4-BDO and HDI at 80 °C for 30 min.

Generally, commercial PU is prepared by multifunctional isocyanate and alcohol. To apply with the results of the model reaction of HDI and BuOH, the reaction behavior of 1,4-butanediol (1,4-BDO) and HDI was examined by FT-IR. In Figure 13, the FT-IR spectra of Figure 13a just-mixed 1,4-BDO and HDI and Figure 13b stirred 1,4-BDO and HDI at 80 °C for 30 min are shown. The carbonyl groups of isocyanates were shown at 2400–2200 cm^−1^. At 80 °C for 30 min, the peak of the reacted urethane C=O was shown at 1800–1600 cm^−1^. The conversion rate was calculated from the peak area ratio of the carbonyl groups of isocyanates at 2400–2200 cm^−1^ and urethane C=O at 1800–1600 cm^−1^.

**Figure 14:**
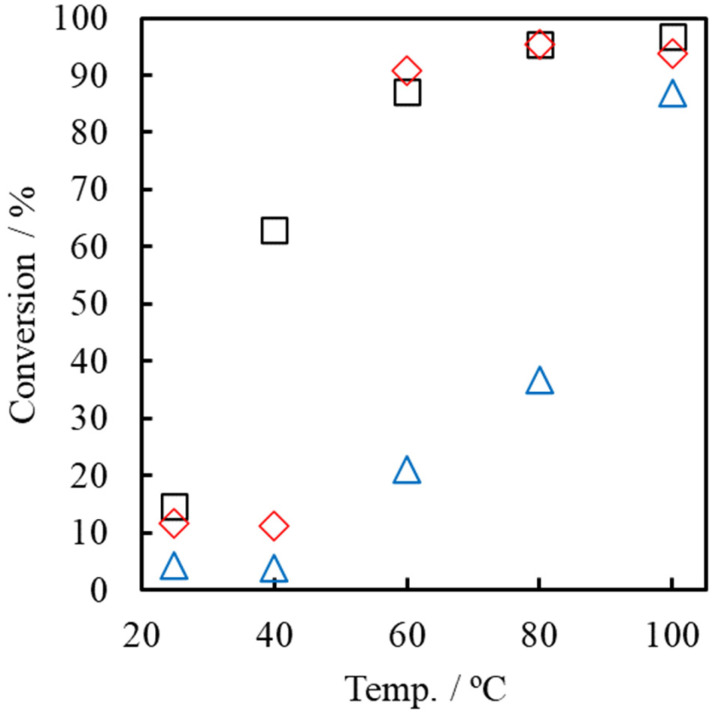
The conversion as a function of temperature during the reaction of 1,4-BDO; 1.0 mmol and HDI; 1.0 mmol with DABCO (□); 0.030 mmol, α-ZrP·DABCO (◇); 17.5 mg, DABCO content: 0.037 mmol, or no catalyst (△); 19.6 mg for 30 min.

Figure 14 shows the conversion of the reaction of 1,4-BDO and HDI after 30 min as a function of temperature during the reaction with DABCO, α-ZrP·DABCO, and no catalyst. The conversions were determined by the FT-IR spectra. In the case of DABCO, the reaction proceeded at 40 °C and the conversion was 63%. For α-ZrP·DABCO, the conversion was 11% at 40 °C. Under heating conditions at 80 °C, the conversions were 95% (DABCO), 96% (α-ZrP·DABCO), and 37% (no catalyst), respectively. The catalyst α-ZrP·DABCO for the reaction of 1,4-BDO and HDI showed high reactivity under heating conditions at 80 °C and was suppressed at 40 °C.

## 3. Materials and Methods

### 3.1. Materials

Zr(HPO_4_)_2_·H_2_O (CZP-100) was purchased from Daiichi Kigenso Kagaku Kogyo Co., Ltd. (Osaka, Japan). 1,4-diazabicyclo[2.2.2]octane (DABCO), hexamethylene diisocyanate (HDI), and butanol were purchased from Tokyo Chemical Industry Co., Ltd. (Tokyo, Japan). Solvents were used as received without further purification.

### 3.2. Measurements

X-ray diffraction (XRD) spectra were recorded using a Rigaku RINT2200 spectrometer (Tokyo, Japan) under the following operating conditions: Cu Kα radiation of 40 kV, 40 mA over a scan range of 3–40° at a rate of 2° min^−1^. NMR spectra were obtained using a JEOL JNM-ECZS spectrometer (400 MHz) (Tokyo, Japan) with tetramethylsilane (TMS) as an internal standard. The ^31^P MAS and ^13^C CPMAS NMR spectra were recorded on a JEOL ECA-600 NMR spectrometer (Tokyo, Japan). Viscosities were measured with an A & D SV-1A tuning fork vibro viscometer using a 2 mL glass vessel (Tokyo, Japan). The morphology images of samples were measured by scanning electron microscopy (SEM) analysis using a JEOL JSM-IT200LA (Tokyo, Japan) at 20 kV working voltage. The samples were prepared using Au (gold)-fine sputtering through a Quick Coater SC-701 (Sanyu Denshi Co., Ltd., Tokyo, Japan) at 10 mA current for 5 min. The Fourier transform infrared spectroscopy (FT-IR) measurements were carried out with an ALPHA spectrometer (Billerica, MA, USA).

### 3.3. Preparation of DABCO-Intercalated α-ZrP (α-ZrP·DABCO)

The intercalation of DABCO into the layers of α-Zr(HPO_4_)_2_·H_2_O (α-ZrP) was carried out by a previously reported method [29]. The composition was Zr(HPO_4_)_2_·0.92DABCO·3.1H_2_O, determined by elemental analysis. E.A. (%), C:12.61, H:4.39, N:5.85.

### 3.4. Typical Procedure for the Reaction of BuOH and HDI with α-ZrP·DABCO

A mixture of BuOH (150 mg, 2.0 mmol) and α-ZrP·DABCO (17.5 mg, DABCO content: 0.037 mmol) was stirred. Subsequently, HDI (176 mg, 1.0 mmol) was introduced into the mixture, followed by heating at 100 °C. After 30 min, a small sample of the reaction mixture was dissolved in CDCl_3_, and its ^1^H-NMR spectrum was obtained to assess the degree of conversion.

### 3.5. Typical Measurements of the Viscosities of BuOH and HDI with α-ZrP·DABCO

A mixture of BuOH (977 mg, 13.2 mmol), HDI (1139 mg, 6.8 mmol), and α-ZrP·DABCO (117 mg, DABCO content: 0.244 mmol) was added to the 2 mL vessel, which was then heated at 80 °C for 80 min. Changes in viscosity as a function of time were measured.

### 3.6. Typical Procedure for the Reaction of 1,4-BDO and HDI with α-ZrP·DABCO

A mixture of 1,4-BDO (92 mg, 1.0 mmol) and α-ZrP·DABCO (17.6 mg, DABCO content: 0.037 mmol) was stirred. Subsequently, HDI (161 mg, 1.0 mmol) was introduced into the mixture, followed by heating at 100 °C. After 30 min, a small aliquot of the reaction mixture was measured by the KBr disc of FT-IR to determine the conversion.

## 4. Conclusions

The catalytic activity of α-ZrP·DABCO in the reaction of HDI and BuOH was investigated. The α-ZrP·DABCO catalysts showed a conversion of 12% at 25 °C; however, the conversion went up to 90% at 80 °C for 30 min. The catalyst α-ZrP·DABCO showed high catalytic activity under the heating conditions, which was confirmed by viscosity measurements, XRD analysis, elemental analysis, ^31^P MAS, and ^13^C CPMAS NMR spectra. After the reaction of HDI and BuOH with α-ZrP·DABCO, the basal distance was decreased from 16.1 Å (2*θ* = 5.4°) for α-ZrP·DABCO to 10.8 Å (2*θ* = 8.2°) at 100 °C, and the urethane compounds were intercalated, confirmed by an elemental analysis and ^13^C CPMAS NMR spectra. The ^31^P MAS NMR spectra showed multiple peaks converted to single peaks at a higher temperature. The ^13^C CPMAS NMR spectra showed the correspondence of urethane products at 60 °C to 100 °C. Interestingly, the XRD patterns of α-ZrP·DABCO treated with BuOH at 40 °C to 100 °C for 30 min showed the disappearance of the peak of 16.1 Å (2*θ* = 5.4°), which was converted to 11.2 Å (2*θ* = 7.9°) due to the deintercalation of DABCO. However, no peak change was observed by α-ZrP·DABCO when treated with HDI. To examine the reactivity of bifunctional monomers, the reaction of 1,4-butanediol (1,4-BDO) and HDI with α-ZrP·DABCO was investigated to show good reactivity at 80 °C and stability at 40 °C. These catalysts can be used for the synthesis of PU as latent thermal catalysts which show stability at ambient temperatures and high activity under heating conditions.

## Figures and Tables

**Table 1:** Composition determined by elemental analysis and basal distance.

Catalyst	C (%)	H (%)	N (%)	*d* (Å)	Composition
α-ZrP·DABCO	12.61	4.39	5.85	16.1	Zr(HPO_4_)_2_·0.92DABCO·3.1H_2_O

**Table 2:** Elemental analyses of α-ZrP·DABCO-RXN.

Sample	C (%)	H (%)	N (%)
α-ZrP·DABCO	12.61	4.39	5.85
BuOH-HDI + α-ZrP·DABCO 40 °C	20.87	4.27	6.93
BuOH-HDI + α-ZrP·DABCO 60 °C	33.08	6.00	8.59
BuOH-HDI + α-ZrP·DABCO 80 °C	29.44	5.45	7.98
BuOH-HDI + α-ZrP·DABCO 100 °C	28.14	5.29	7.78

**Table 3:** Elemental analyses of α-ZrP·DABCO treated with BuOH at various temperatures for 30 min.

Sample	%-C	%-H	%-N
α-ZrP·DABCO	12.61	4.39	5.85
BuOH + α-ZrP·DABCO 40 °C	13.46	2.79	5.20
BuOH + α-ZrP·DABCO 60 °C	12.43	2.68	4.75
BuOH + α-ZrP·DABCO 80 °C	12.51	2.56	4.80
BuOH + α-ZrP·DABCO 100 °C	12.31	2.39	4.73

## Data Availability

Data are contained within the article and Supplementary Materials.

## Supplementary Materials

The following supporting information can be downloaded at: https://www.mdpi.com/article/10.3390/molecules29235569/s1, Figure S1: FT-IR spectrum of commercial α-ZrP [30]. Figure S2: FT-IR spectrum of α-ZrP·DABCO. Figure S3: FT-IR spectrum of the typical reaction of HDI and BuOH with DABCO at 100 °C for 30 min. Figure S4: TGA curve of α-ZrP·DABCO.
