# Supplementary material for: DABCO-Intercalated α-Zirconium Phosphate as a Latent Thermal Catalyst in the Reaction of Urethane Synthesis
Source: Molecules. 2024 Nov 25;29(23):5569. doi: 10.3390/molecules29235569 (PMC11643932; doi:10.3390/molecules29235569)
Supplement: Supplementary file 1 [file molecules-29-05569-s001.zip › molecules-3274482-supplementary.pdf]

DABCO-Intercalated α-Zirconium Phosphate as a Latent Thermal Catalyst in the Reaction of Urethane Synthesis

Osamu Shimomura [1,*], Yushi Arisaka [1], Astrid Rahmawati [1], Shekh Md. Mamun Kabir [1,2], Motohiro Shizuma [3] and Atsushi Ohtaka [1]

[1] Department of Applied Chemistry, Osaka Institute of Technology, 5-16-1 Omiya, Ashahi-ku, Osaka 535-8585, Japan
[2] Department of Wet Process Engineering, Bangladesh University of Textiles, Tejgaon, Dhaka-1208, Bangladesh
[3] Osaka Research Institute of Industrial Science and Technology, 1-6-50 Morinomiya, Joto-ku, Osaka 536-8553, Japan
* Correspondence: osamu.shimomura@oit.ac.jp; Tel.: +81-6-6954-4269

1) Figure S1. FT-IR spectrum of commercial α-ZrP.
2) Figure S2. FT-IR spectrum of α-ZrP·DABCO.
3) Figure S3. FT-IR spectrum of the typical reaction of HDI and BuOH with DABCO at 100 °C for 30 min.
4) Figure S4. TGA curve of α-ZrP·DABCO.

Mesurements

Thermogravimetric analysis (TGA) was carried out with Netzsh TG-DTA2000SA (Selb, Germany) at a heating rate of 10/min under nitrogen.

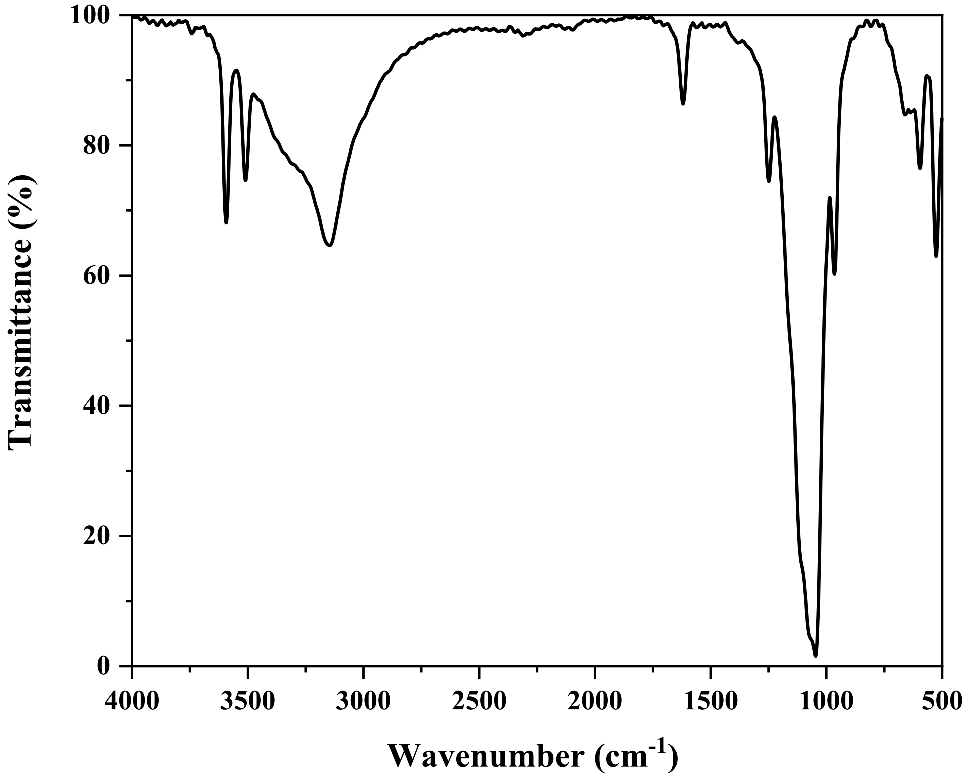

Figure S1. FT-IR spectrum of commercial α-ZrP.

Figure S1 presents the FT-IR spectrum of commercial α-ZrP (CZP-100). The absorption bands associated with O-H stretching vibration were observed at 3592 and 3512 cm^{-1}. The absorption peak at 1620 cm^{-1} is the deformation vibrations of the O-H bonds of the water molecules. The ν P-O was observed at 1041 cm^{-1} [1].

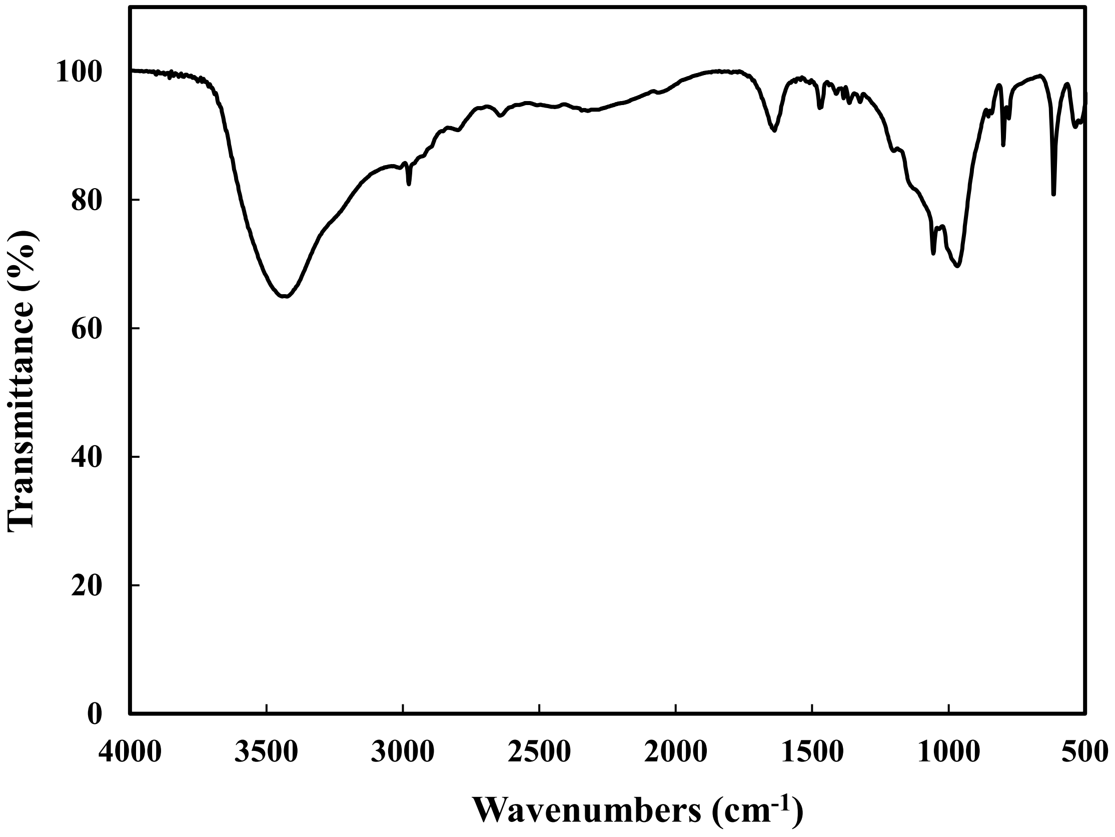

Figure S2. FT-IR spectrum of α-ZrP·DABCO.

Figure S2 shows the FT-IR spectrum of α-ZrP·DABCO. The characteristics peaks of C-H stretching and C-H bending of DABCO were observed at 2958-2900 and 1465 cm^{-1}, respectively. The major peak of α-ZrP was observed at 1041 for ν P-O. All spectra confirmed the successful synthesis of α-ZrP·DABCO.

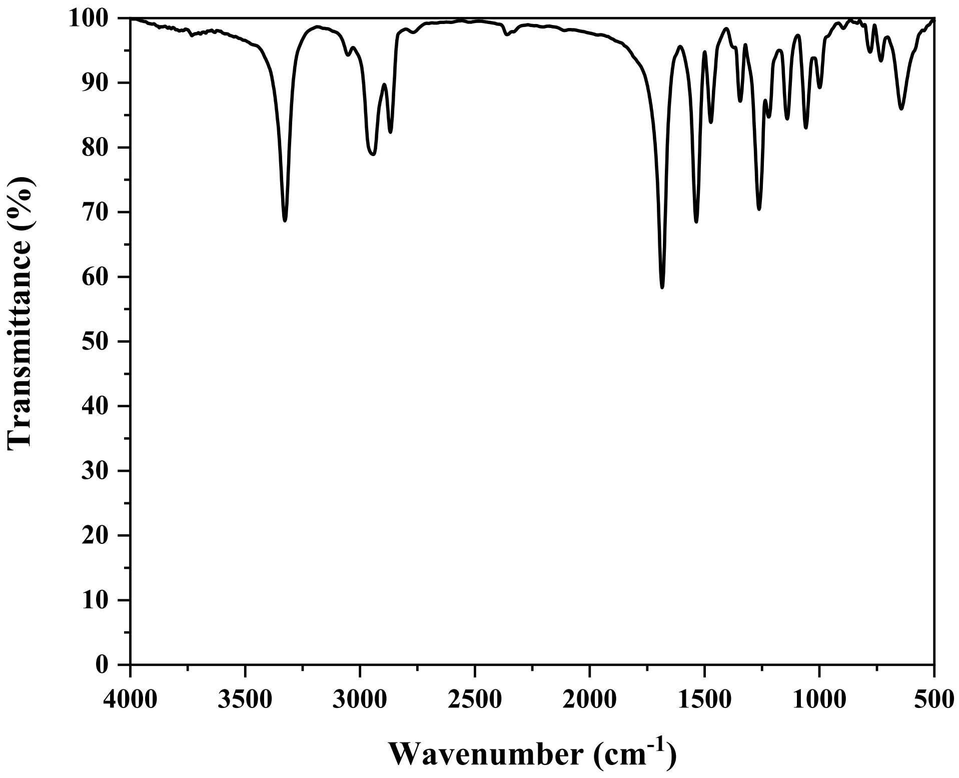

Figure S3. FT-IR spectrum of the typical reaction of HDI and BuOH with DABCO at 100 °C for 30 min.

Figure S3 shows the FT-IR spectrum of typical reaction of HDI and BuOH with DABCO. The absorption bands at 3326 cm^{-1} belong to the N-H stretching of the urethane product. The absorption bands of the stretching of C-H at 2944 and 2866 cm^{-1} and of C=O at 1686 cm^{-1} were observed, respectively.

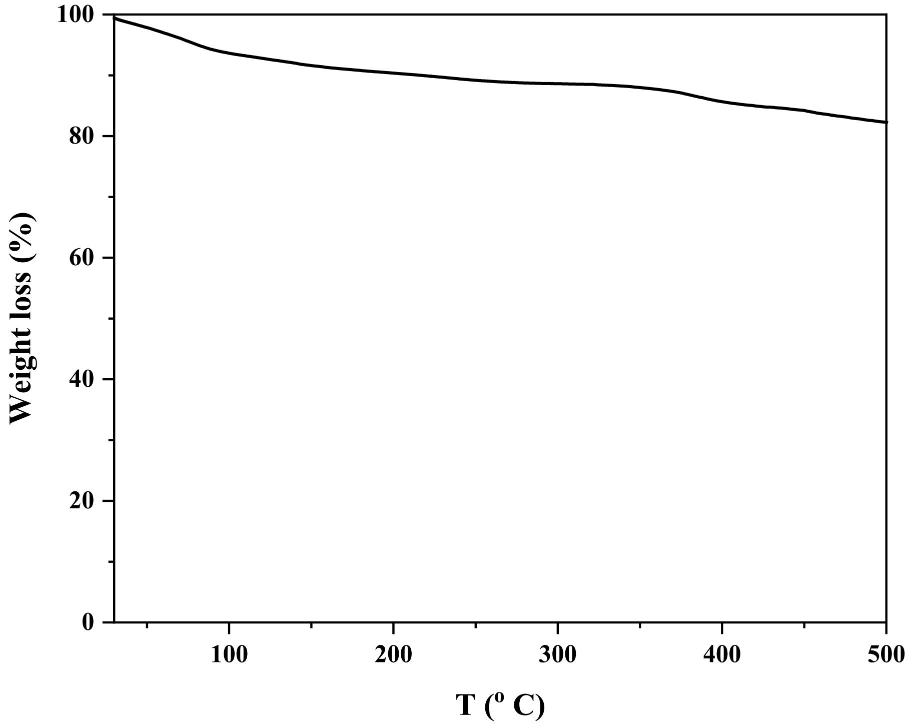

Figure S4. TGA curve of α-ZrP·DABCO.

The thermogravimetric analysis curve in Figure S4 shows a weight loss between 28 and 500 ºC. The weight loss of 6.42% at 100 ºC was attributed to the release of water molecules. A total weight loss of 17.73% may correspond to the thermal decomposition of organic compounds.

Reference

1. Garcia, M. E.; Naffin, J. L.; Deng, N.; Mallouk, T. E., Preparative-Scale Separation of Enantiomers Using Intercalated α-Zirconium Phosphate. *Chem. Mater.* **1995,** *7*, 1968–1973. https://doi.org/10.1021/cm00058a030
